# Bone Fracture Acute Phase Response—A Unifying Theory of Fracture Repair: Clinical and Scientific Implications

**DOI:** 10.1007/s12018-018-9256-x

**Published:** 2018-12-29

**Authors:** Courtney E. Baker, Stephanie N. Moore-Lotridge, Alexander A. Hysong, Samuel L. Posey, J. Patton Robinette, Deke M. Blum, Michael A. Benvenuti, Heather A. Cole, Satoru Egawa, Atsushi Okawa, Masanori Saito, Jason R. McCarthy, Jeffry S. Nyman, Masato Yuasa, Jonathan G. Schoenecker

**Affiliations:** 1 Department of Orthopaedics, Mayo Clinic, 200 1st Ave SW, Rochester, MN 55903 USA; 2 Department of Orthopaedics and Rehabilitation, Vanderbilt University Medical Center, 1215 21st Ave. South, Suite 4200 MCE, South Tower, Nashville, TN 37232 USA; 3 Department of Pathology, Microbiology, and Immunology, Vanderbilt University Medical Center, 1161 21st Ave. South, Nashville, TN 37232 USA; 4 Vanderbilt University School of Medicine, 1161 21st Ave S, #D3300, Nashville, TN 37232 USA; 5 Department of Orthopaedic Surgery, Tokyo Medical and Dental University, Yushima Bunkyo Ward, Tokyo, 113-8519 Japan; 6 Masonic Research Institute, 2150 Bleecker St, Utica, NY 13501 USA; 7 Department of Biomedical Engineering, Vanderbilt University, PMB 351631, 2301 Vanderbilt Place, Nashville, TN 37235 USA; 8 Department of Veterans Affairs, Tennessee Valley Health Care System, F-519 VA Acre Building, 1210 24th Ave. South, Nashville, TN 37232 USA; 9 Department of Pharmacology, Vanderbilt University, 2200 Pierce Ave, Robinson Research Building, Nashville, TN 37232 USA; 10 Department of Pediatrics, Vanderbilt University Medical Center, 4202 Doctor’s Office Tower, 2200 Children’s Way, Nashville, TN 37232 USA

**Keywords:** Fracture repair, Fracture vascularity, Strain, Acute phase response, Endochondral ossification, Non-union

## Abstract

Bone fractures create five problems that must be resolved: bleeding, risk of infection, hypoxia, disproportionate strain, and inability to bear weight. There have been enormous advancements in our understanding of the molecular mechanisms that resolve these problems after fractures, and in best clinical practices of repairing fractures. We put forth a modern, comprehensive model of fracture repair that synthesizes the literature on the biology and biomechanics of fracture repair to address the primary problems of fractures. This updated model is a framework for both fracture management and future studies aimed at understanding and treating this complex process. This model is based upon the fracture acute phase response (APR), which encompasses the molecular mechanisms that respond to injury. The APR is divided into sequential stages of “survival” and “repair.” Early in convalescence, during “survival,” bleeding and infection are resolved by collaborative efforts of the hemostatic and inflammatory pathways. Later, in “repair,” avascular and biomechanically insufficient bone is replaced by a variable combination of intramembranous and endochondral ossification. Progression to repair cannot occur until survival has been ensured. A disproportionate APR—either insufficient or exuberant—leads to complications of survival (hemorrhage, thrombosis, systemic inflammatory response syndrome, infection, death) and/or repair (delayed- or non-union). The type of ossification utilized for fracture repair is dependent on the relative amounts of strain and vascularity in the fracture microenvironment, but any failure along this process can disrupt or delay fracture healing and result in a similar non-union. Therefore, incomplete understanding of the principles herein can result in mismanagement of fracture care or application of hardware that interferes with fracture repair. This unifying model of fracture repair not only informs clinicians how their interventions fit within the framework of normal biological healing but also instructs investigators about the critical variables and outputs to assess during a study of fracture repair.

## Significance

### The Need for a Complete Understanding of Fracture Repair in Orthopedics

More than 16 million fractures are treated in the United States each year [1, 2]. Up to 10% of these are complicated by delayed union or non-union, which result in significant patient morbidity and economic burden on our healthcare system [1, 2]. Critical to addressing this public health concern is understanding both the clinical interventions and physiological processes involved in fracture repair.

There has been enormous growth in the scientific understanding of fracture healing over the last century. This has led to advances in both clinical practice and technology that have improved patient outcomes. With this rapid expansion, however, comes a large body of knowledge that has been difficult to synthesize into a modern, comprehensive theory of fracture repair.

The goal of this review is to integrate the most significant advancements in fracture biology to create a coherent and unified theory of fracture repair. The most direct way to review the complicated process of fracture repair is through the body’s systematic process of healing itself: the acute phase response (APR). To do so, this review focuses on the primary problems created by a fracture and relates each of these problems to specific, well-recognized complications. It then provides a thorough explanation of the body’s biologic response to resolve these problems and prevent complications. Finally, it uses what is currently known about the biology of fracture repair to explain when and how clinicians should intervene to improve patient outcomes.

## Introduction

### The Primary Problems Created by Fractures

**Fig. 1:**
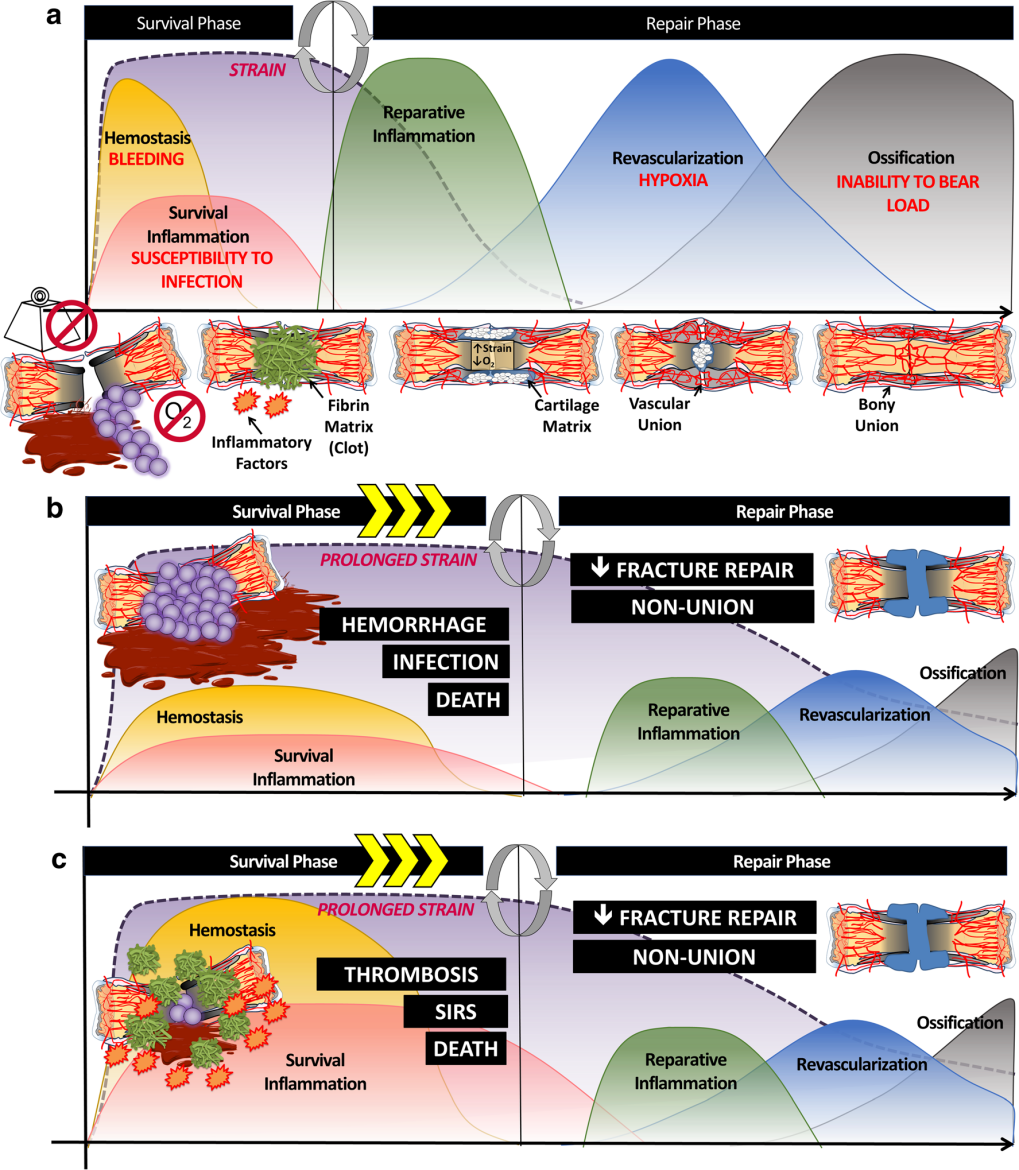
The body’s response to fracture injury: the acute phase response (APR). **a** Following a fracture, the body must resolve 5 primary problems: bleeding, susceptibility to infection, disproportionate strain, bone hypoxia, and inability to bear weight. The APR is the body’s hormonal response system to injury. The APR first resolves lethal problems such as bleeding and susceptibility to infection in the “survival phase,” then transitions to the “repair phase” where strain is reduced by cellular and acellular factors allowing new vasculature to extend across the fracture site, reducing bone hypoxia, and leading to vascular union. **b** If the APR is insufficient or **c** inappropriately exuberant, complications, or “villains,” arise, such as hemorrhage, infection/sepsis, deep vein thrombosis (DVT), SIRS, and, in severe cases, death. Complications that prolong the survival phase will delay the initiation of repair

Fractures create five primary problems: bleeding, susceptibility to infection, disproportionate interfragmentary strain, bone hypoxia, and an inability to bear weight (Fig. 1). First, bleeding occurs due to bone’s open vascular system, which makes rapid hemostasis a challenge following a fracture. Second, infection is a common concern as fractures disrupt the body’s protective anatomical compartments. Third, strain, defined as the change in length of a fracture gap upon loading relative to its overall length when unloaded, can be detrimental to fracture healing if it is disproportionate to the intended ossification process. Fourth, bone hypoxia occurs as fractures result in both bony and vascular discontinuity, resulting in a large area of under-perfused, hypoxic bone tissue. Finally, the inability to bear a load must be resolved before a fracture is considered healed. After achieving vascular and bone union, the bone begins the long process of remodeling to a structurally and energetically efficient construct. In order to return to pre-injury function, a bone must not only physically bridge the fracture gap, but also be able to transmit force across it, ideally without altered joint mechanics.

### Acute Phase Response—The “Heroes”

In order to address the five primary problems created by fractures, the body utilizes the APR, a complex hormonal system for surviving injury and repairing damaged tissues following any trauma, including fractures (Fig. 1a). The APR occurs in an orderly fashion and is divided into two distinct phases—termed “survival” and “repair.” The “survival” phase functions to contain the injury by utilizing coagulation and inflammation to achieve hemostasis and prevent infection. These are the most immediate, lethal threats of a fracture and must be addressed quickly. Once the body has survived these problems, it then proceeds to the “repair” phase, which aims to recreate functional anatomy by minimizing excessive strain at the fracture site, restoring vascular unity, and repairing bony anatomy to restore load-bearing ability.

### Complications of Fracture Repair—The “Villains”

If stimulated appropriately, the APR heals a fracture without incident. If, however, the APR is insufficient (Fig. 1b) or inappropriately exuberant (Fig. 1c), significant complications, or “villains,” of fracture repair can occur, such as hemorrhage, thrombosis, systemic inflammatory response syndrome (SIRS), infection, death, and/or impaired bony union.

These complications can occur either in the “survival phase” (bleeding, thrombosis, SIRS, infection, death) or “repair phase” (impaired fracture healing). In accordance with the orderly nature of the APR, complications that prolong the “survival phase” will inevitably delay the initiation of the “repair phase” (Fig. 1b, c). Therefore, it is important to consider that a dysfunctional “survival phase” or “repair phase” can both result in the same pathology—impaired fracture repair.

In order to resolve problems of fracture repair, identifying the cause of the pathophysiology must first be determined. Without a comprehensive understanding of the many pathologic causes of impaired fracture repair, it can be difficult to identify the appropriate treatment.

## The Acute Phase Response

### Survival Phase: Contain the Injury

The human body is organized into discrete functional anatomical compartments. When a fracture occurs, the normal architecture and vasculature of the bone, periosteum, and surrounding soft tissues are disrupted [3–5]. When these compartments are disrupted, damaged cells release cytokines that travel to hepatocytes and stimulate thousands of gene transcripts that upregulate coagulative, inflammatory, reparative, and angiogenic factors that stimulate the APR which begins to address the principal problems of the injury. As mentioned previously, the first component of this response is survival from the most life-threatening complications of tissue damage: hemorrhage and infection**.**

Bone is a highly vascular organ receiving roughly 10–15% of the heart’s total cardiac output via a combination of metaphyseal/epiphyseal, diaphyseal, and periosteal arteries. These arteries contribute to the open-sinusoidal vascular network that supplies both cortical and medullary bone [6, 7], and fracture-induced disruption of these vessels poses a serious bleeding risk. Several studies estimate that blood loss from an isolated closed femoral shaft fracture can exceed 1 L, posing a high risk for hypovolemic shock [8, 9].

In addition to hemorrhage, the disruption of protective tissue barriers secondary to a fracture increases a patient’s risk for the development of serious infections. Skin, for example, a major component of the body’s innate immune system, is often breached in traumatic long bone fractures. This allows bacterial flora to directly inoculate the fracture site, greatly increasing the risk of infection [10]. Infection rates of greater than 30% have been reported following surgically treated open tibial fractures [11]. Closed fractures can also result in infection due to the tropism of bacteria for damaged and repairing musculoskeletal tissue and subsequent hematogenous seeding [12]. Ongoing bodily infection exposes the body to a continuous state of tissue damage and results in hyperinflammation. If severe enough, this continuous infection and hyperinflammation can lead to devastating complications such as thromboembolism, sepsis, disseminated intravascular coagulopathy, and multiple organ failure [13].

To survive hemorrhage and infection in the setting of a fracture, the body’s first task is to achieve hemostasis. Thus, the survival portion of the APR begins with reactive contraction of arterioles followed by activation of the coagulation cascade secondary to exposure of subendothelial collagen, resulting in the formation of an intravascular and predominantly extravascular meshwork rich in platelets and fibrin [3, 14, 15]. This meshwork, commonly known as the fracture hematoma, acts to both resolve bleeding at the fracture site and to contain and eliminate potential sources of infection [12, 16, 17]. Specifically, fibrin has been shown to physically trap bacteria at the site of tissue damage, preventing their dissemination [12, 13, 18]. Within the fracture hematoma, a complex milieu of chemotactic factors released from platelets, complement factors, proinflammatory cytokines released from necrotic tissue, and integrin expression on fibrin together act to attract inflammatory cells to the fracture site which is essential for healing [3, 12, 13, 19, 20]. Neutrophils are the predominant inflammatory cell in the 24 hours immediately following a fracture, acting to further contain and destroy pathogens at the fracture site [3, 12, 13]. Multiple studies have shown the importance of fracture hematoma to healing in a variety of fracture models—removal of the hematoma up to 4 days post fracture resulted in delayed or non-union in an animal model [21–23].

### Repair Phase: Reconstruct the Bone

**Fig. 2:**
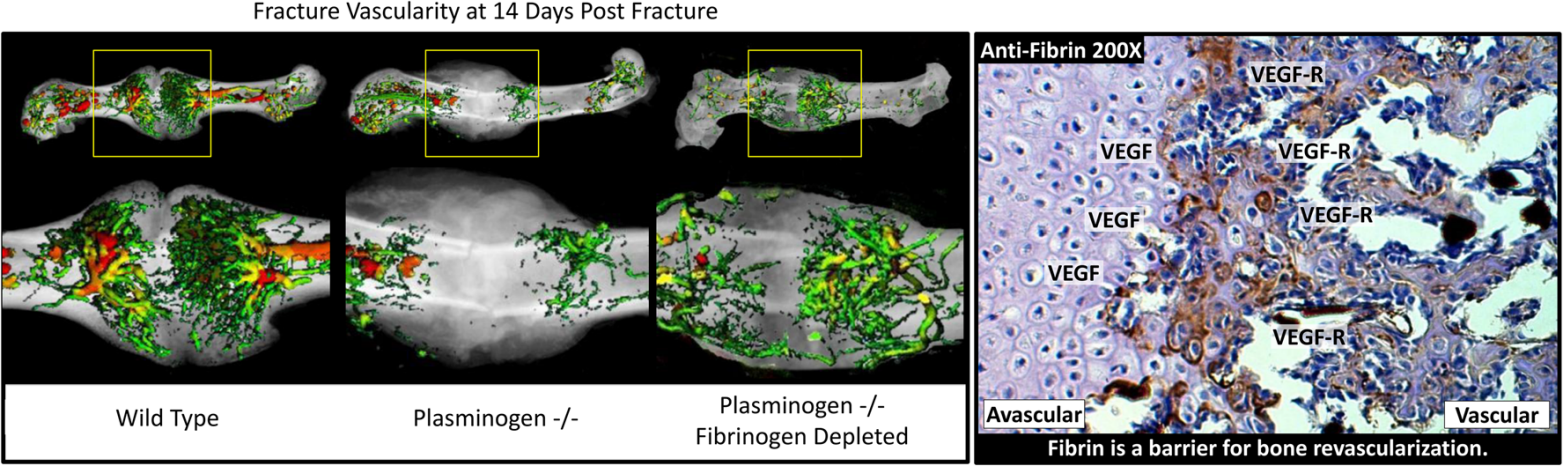
Fibrin must be removed following fracture injury for revascularization and subsequent ossification to occur. Wild-type mice exhibit robust vascularity at the fracture site 14 days post fracture. In plasminogen knockout mice, fibrin cannot be removed from fracture site, and angiogenesis is significantly inhibited. When fibrinogen was depleted in plasminogen knockout mice, angiogenesis is largely restored. Fibrin acts as a barrier for bone revascularization, preventing VEGF produced by the hypertrophic chondrocytes from effectively reaching the VEGF receptor on endothelial cells. This demonstrates the importance of completely resolving one phase of repair before a next can begin

After hemostasis has been achieved and susceptibility to infection has been resolved, the APR shifts from the survival phase to the repair phase, beginning with the removal of both necrotic tissues and the provisional fibrin matrix in preparation for vascular invasion and subsequent ossification [3, 12, 13]. As the protease plasmin degrades fibrin clot within the fracture hematoma, neutrophils present at the fracture site begin to attract the second wave of inflammatory cells, macrophages [3, 17, 24]. Macrophages consume the necrotic tissue and fibrin split products that are contained within the fracture site, and secrete chemotactic factors that promote the recruitment of mesenchymal stem cells and osteoprogenitor cells to the fracture site in order to begin tissue regeneration [3–5]. The transition of macrophages from “M1” to “M2” phenotype has been associated with the switch from a pro-inflammatory function to a pro-reparative function, and this macrophage transition parallels the shift between the “survival” and “repair” phases of the APR [25]. Failure to effectively remove fibrin from the fracture site impedes angiogenesis, thus preventing ossification (Fig. 2) [17, 26]. This exemplifies a central tenet of the APR: in order for proper tissue healing to occur, the body must successfully complete one phase before it can transition to the next [12].

At this point, the healing fracture still faces three remaining problems: unresolved strain, bone hypoxia, and an inability to bear weight. The resolution of each problem occurs in a chronological and co-dependent manner. First, excessive interfragmentary strain is resolved through the synthesis of a chondroid soft tissue callus. After sufficient reduction of strain, chondrocytes promote angiogenesis into the soft callus. Vascular growth then provides the nutrients and progenitor cells needed to form stable bone, thus restoring the bone’s ability to bear weight (Fig. 1a). As the ability to bear weight returns, the bony hard callus begins to remodel in response to load-bearing forces, resulting in a transition from woven to lamellar bone and a reduction in hard callus volume [27].

**Fig. 3:**
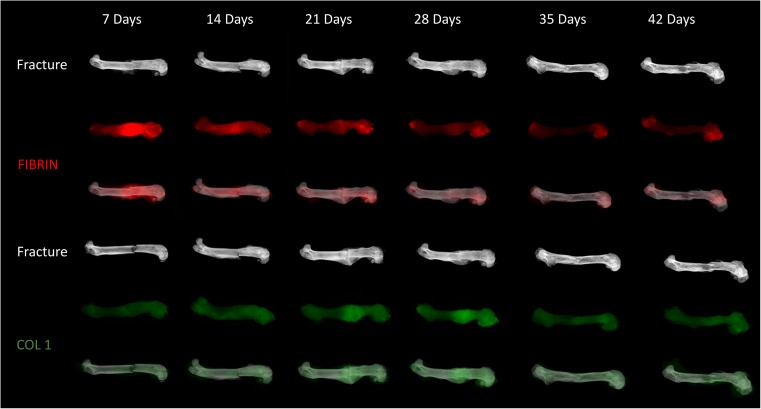
Matrix evolution at the fracture site. The evolution of matrices is shown in a murine model of a transverse femur fracture with fixation. By 14 days post fracture, the fluorescently labeled fibrin matrix (red) has begun to be cleared and type 2 collagen and type 10 collagen (not pictured fluorescently) begin to form at the fracture site. By 21 days post fracture, the presence of type 1 collagen (green) is pronounced at the site of fracture healing in the form of hard callus. As remodeling of the hard callus occurs through 42 days post fracture, there is a decrease in observable type 1 collagen signal

In a broad sense, fracture healing can be considered an evolution of matrices. While the predominant matrix of the survival phase is fibrin, the ultimate goal of repair is to first remove the temporary fibrin matrix, promote vascular invasion, and allow for ossification of type 1 collagen (Fig. 3). While each of these processes must occur in series, they are not independent of one another, such that the cells that mediate each step are specifically designed to promote the next phase of healing. A thorough understanding of these cells and their respective roles, as discussed next, will help clinicians prevent complications and facilitate timely fracture repair in their patients.

## Coordinated Teams of Cells in the Repair Phase

**Fig. 4:**
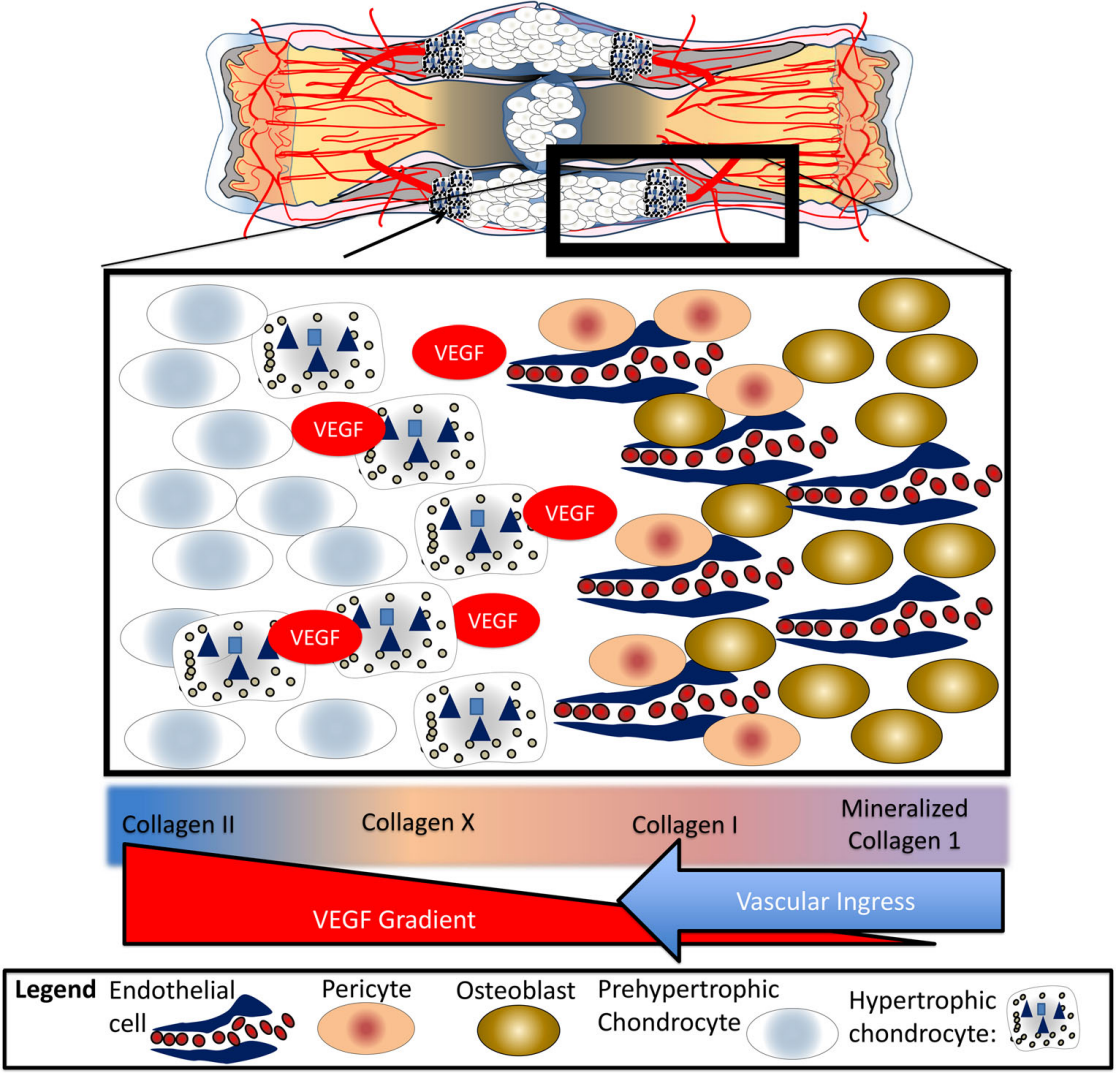
Cells in the fracture callus. Following a fracture, pre-hypertrophic chondrocytes function to resolve strain by producing a biomechanical matrix composed of cellular and acellular materials, primarily collagen 2. Once the pre-hypertrophic chondrocytes have sufficiently minimized strain, they hypertrophy, becoming hypertrophic chondrocytes that will provide vascular endothelial growth factor (VEGF) to attract endothelial cells, and promote ossification in conjunction with osteoblasts by producing bone morphogenic protein (BMP) and nanohydroxyapatite

Following removal of the provisional fibrin matrix, the repair phase of the APR is characterized by teams of cells that work to address strain, hypoxia, and inability to bear weight following a fracture. Depending on the microenvironment of the fracture, these teams of cells heal fractures through either intramembranous and/or endochondral ossification [28]. Understanding the differences between these processes starts with distinguishing the major cell types at work: progenitor cells, pre-hypertrophic chondrocytes, hypertrophic chondrocytes, endothelial cells, and osteoblasts (Fig. 4).

Chondrocytes are unique in that they thrive in hypoxic environments. They play a critical role in fracture healing, much like they do at the physis, by providing biomechanical support and directing vascular ingress and subsequent ossification (Fig. 4) [28, 29]. The main function of the pre-hypertrophic chondrocyte is biomechanical, such that in response to strain, these cells create an organized, force-absorbing extracellular matrix composed predominantly of type II collagen [30, 31]. These same cells are also present in healthy articular cartilage, a physiologically hypoxic environment, where they function to absorb shock at the joint surface [32]. Cell proliferation and type 2 collagen matrix production continues at the fracture site until strain is reduced to a degree that allows fracture healing to progress [16, 33].

Once interfragmentary strain has been sufficiently reduced, pre-hypertrophic chondrocytes begin to hypertrophy and progress to their second form, the hypertrophic chondrocyte, which functions to promote vascularity and create a supportive environment for osteoblasts. With strain resolved, hypertrophic chondrocytes are free to produce cytokines and growth factors for vascular ingress and ossification. The most recent literature proposes that these cells release microvesicles coated with alkaline phosphatase (ALP) to counter pyrophosphate, the principle inhibitor of hydroxyapatite formation [34, 35]. These microvesicles are filled with nanohydroxyapatite, a potent seed crystal for micro-hydroxyapatite [36]. Together with type 10 collagen, these products promote the calcification of type 2 collagen, an important precursor of bone. The microvesicles also contain concentrated vascular endothelial growth factor (VEGF) and bone morphogenic protein-2 (BMP-2), needed to promote angiogenesis and osteogenesis, respectively (Fig. 4) [16, 37].

Endothelial cells manage the transition from chondroid soft tissue callus to hard callus. Unlike vasculature found in the rest of the body, endothelial cells do not possess smooth muscle and are unable to withstand significant strain. Thus, similar to hypertrophic chondrocytes, they rely on pre-hypertrophic chondrocytes to resolve strain prior to their arrival. Once strain is resolved, endothelial cells are drawn into the soft tissue callus by the VEGF produced by the hypertrophic chondrocytes (Fig. 4) [38, 39].

Osteoblasts, or the main bone forming cell, are intricately linked to endothelial cells as they migrate into the soft tissue callus. Once within a bone-forming microenvironment, the osteoblast produces type 1 collagen, hydroxyapatite, BMP-2, and additional VEGF, encouraging further revascularization and continuing the ossification process by promoting the deposition of micro-hydroxyapatite on type I collagen [31, 40]. Mineralizing of type 1 collagen continues until the osteoblast has surrounded itself with hydroxyapatite, thus becoming an osteocyte, or undergoes apoptosis [35].

The precise cellular origin of the chondroblasts and osteoblasts during fracture repair remains elusive. Classically, it has been suggested that during endochondral ossification, osteoblasts develop from osteoprogenitors brought in with angiogenic vasculature to replace the apoptotic chondrocytes [30, 32, 33]. This is supported by modern data which links osteoblast precursor invasion with blood vessels and with apoptosis of chondrocytes [41].

Recent studies have suggested that there are two dominant sources of progenitor cells for fracture repair: periosteum and endosteum. Lineage-tracing experiments have demonstrated that a subset of periosteal stem cells serve as sources of chondrocytes and osteoblasts during fracture repair [42], while bone marrow stem cells (BMSCs) seem to undergo osteoblast differentiation exclusively [43, 44]. Furthermore, recent data also suggests that pericytes and BMSCs function as critical sources of trophic factors for directing differentiation [43, 45, 46]. In addition to providing new vessels and coordinating differentiation of other stem cells, pericytes themselves have also been shown to retain capacity for mesenchymal differentiation into multiple tissue types including osteoblast or chondrocyte precursors [47–49]. Taken together, many studies have suggested multiple cell types within the musculoskeletal system poses the capacity to become osteocytes or chondrocytes.

To add even more complexity, recent advanced animal models and cell-lineage tracking methodologies have suggested that osteoblasts can also develop directly from chondrocyte precursors through a process of trans-differentiation, promoted by endothelial factors [36, 50]. While a significant advancement of our understanding, this intriguing concept is still consistent with the classic teaching that osteoblast development at the fracture site is spatially and temporally predicated on vascular ingress to the site of hypertrophic chondrocytes, and reinforces that hypoxic signaling and vascularity are absolutely essential to fracture healing. The specific functions and mechanisms of various cell types for providing progenitors versus directing differentiation are still being sorted, but the pre-eminent roles of the periosteum, endosteum, and vasculature in these processes are firmly established.

Once bony union has been established across a fracture site, a coordinated catabolic and anabolic effort between osteoclasts, osteoblasts, and vasculature is responsible for the long process of revising the irregular woven bone of the fracture callus into structurally efficient lamellar bone to restore cortical structure and a medullary canal [51]. This process is driven by high levels of IL-1 and TNF-α as well as mechanical loading, and can take years for complete resolution [52–54]. The cell most associated with hard callus remodeling is the osteoclast. Studies on osteoclast-deficient mice and mice treated with osteoprotegrin or bisphosphonates—potent inhibitors of osteoclastogenesis and osteoclast function, respectively—showed minimal effect on fracture union but significant inhibition of hard callus remodeling [55–59]. While woven bone in the hard tissue callus is structurally less efficiently compared to remodeled lamellar bone, it has a larger cross-section making the overall construct similar in mechanical properties [55, 57]. This matches the low incidence of complications stemming from delayed fracture remodeling in otherwise healthy individuals. There is still conflicting opinion whether inhibiting osteoclast-mediated hard callus remodeling can be beneficial in some circumstances, particularly its role for treating fractures in osteoporotic individuals. The inability to produce significantly more dense bone by simply blocking osteoclasts underscores the linked nature of anabolism and catabolism during remodeling.

Innovation opportunity: A quantifiable, in vivo measurement of cellular “biological potential.” Clearly, fracture repair requires properly functioning progenitor cells. Currently, there is no direct measure of this “biological potential” and is instead determined by clinical intuition. As an example, orthopedic surgeons approach a femoral neck fracture differently in a 30-year-old as opposed to an 80-year-old as their clinical intuition indicates that the biological potential is vastly different between these patients. A laboratory test that provides a measure of a patient’s “biological potential” would be invaluable in clinical decision-making.

### The Ossification Processes—Strain, Vascularity, and the Significance of a Chondrocyte Intermediate

**Fig. 5:**
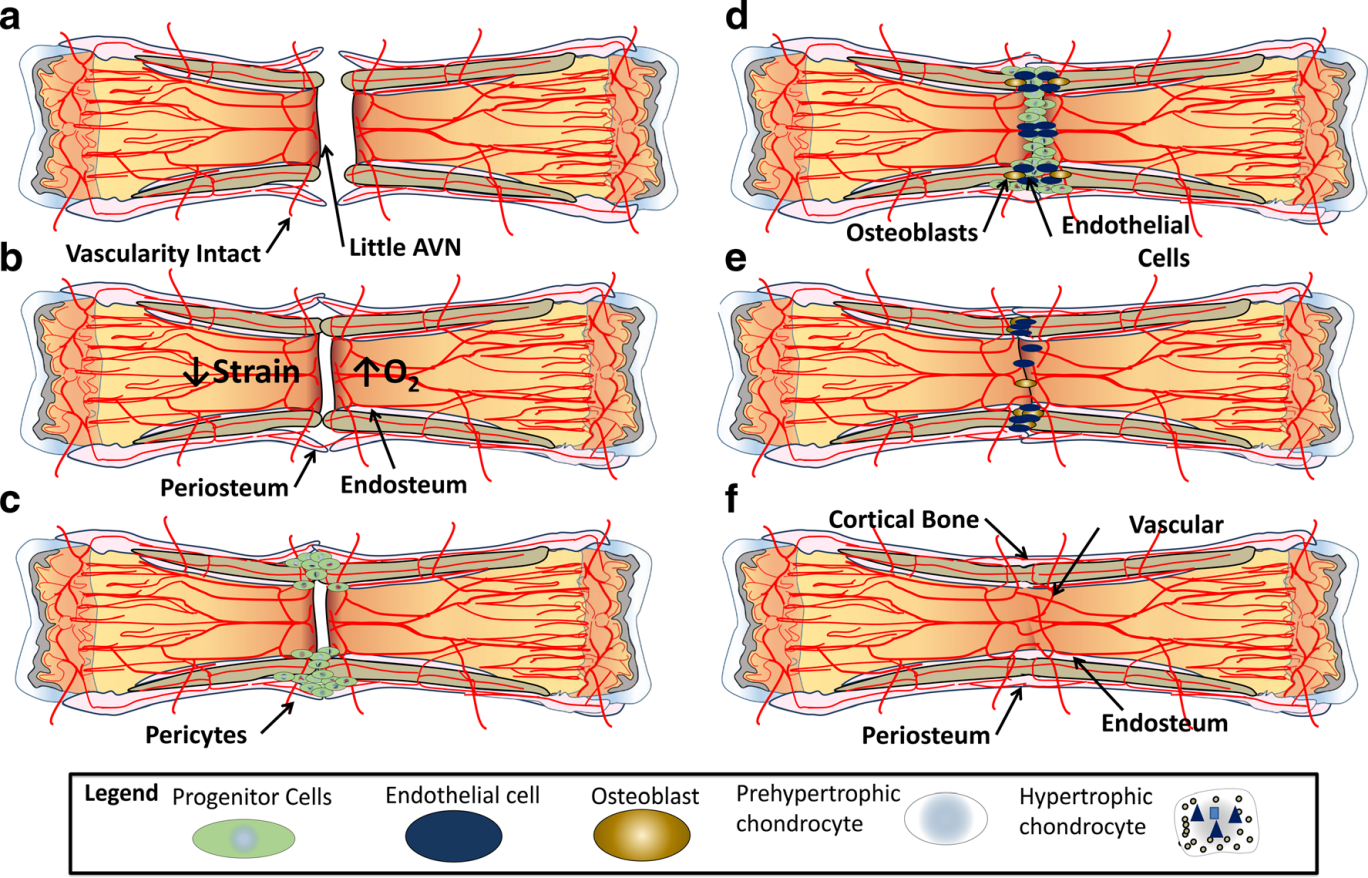
Intramembranous ossification/primary bone healing. In fractures with (**a**) intact vascularity, little avascular necrosis, (**b**) low strain, good oxygenation, and healthy periosteum and endosteum, intramembranous ossification or primary bone healing is possible. Progenitor cells invade the fracture site (**c**) and are followed by (**d**) endothelial cells and subsequently transition directly into osteoblasts. The osteoblasts (**e**) gradually achieve bony union that includes (**f**) union of cortical bone, intramedullary vascularity, periosteum, and endosteum

**Fig. 6:**
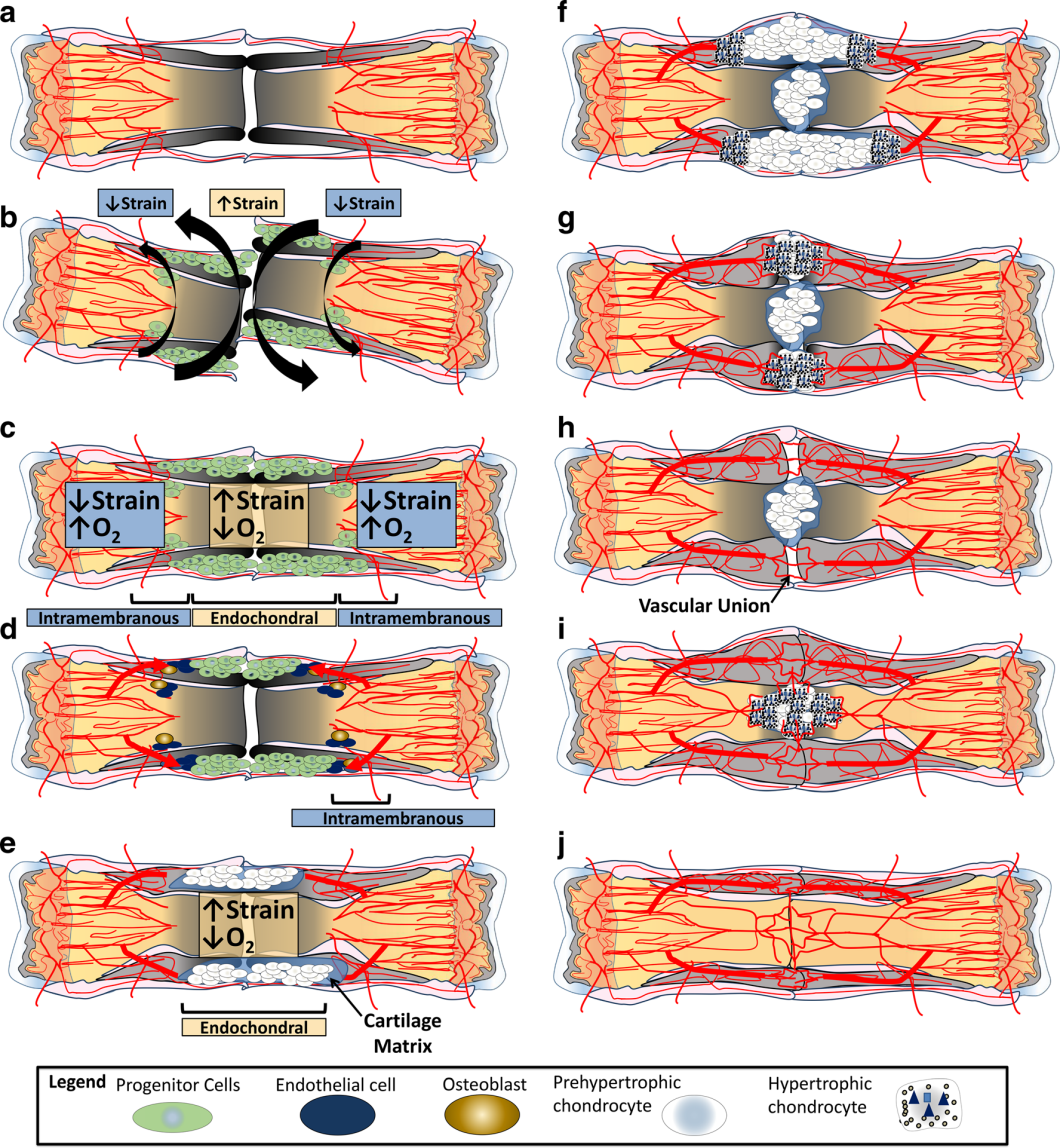
Secondary bone healing. In the majority of fractures, the structural integrity of the bone and the vascular supply to the fracture site are disrupted, leading to (**a**) hypoxia and interfragmentary motion. Under these conditions, (**b**) progenitor cells are drawn to the fracture site and, depending on the conditions of strain and oxygen tension, either (**c**) intramembranous or endochondral ossification will ensue. (**d**) At the periphery of the fracture (relatively preserved oxygen supply and low strain), progenitor cells in close association with the bone’s intact blood supply differentiate into osteoblasts and begin the process of intramembranous ossification. Within the center of the fracture site (high strain and low oxygen tension) (**e**), the progenitor cells develop into pre-hypertrophic chondrocytes, proliferate in response to strain, and resolve strain by forming a biomechanical extracellular matrix. When strain is sufficiently resolved, (**f**) these chondrocytes undergo hypertrophy and become hypertrophic chondrocytes that direct angiogenesis and osteogenesis. (**g**) Hypertrophic chondrocytes promote vascular invasion and osteogenesis by releasing BMP, VEGF, and hydroxyapatite. (**h**) Vascular union always precedes bony union at the fracture site, as the endothelial cells are necessary for ossification. (**i**) With bony union of the fracture callus, the fracture is stabilized, and the remaining chondrocytes become hypertrophic. (**j**) The fracture is now healed and remodeling proceeds

There are two methods of bone formation: intramembranous and endochondral ossification. Intramembranous ossification (Fig. 5) is the formation of bone without a cartilage intermediate and occurs when osteoblasts form bone on an existing connective tissue matrix. Endochondral ossification (Fig. 6) is bone formation with a cartilage intermediary. As described above, the purpose of the cartilage intermediate is to resolve strain (pre-hypertrophic chondrocyte) and provide a stimulus for vascular ingress and subsequent ossification (hypertrophic chondrocytes). Fractures that have a significant amount of interfragmentary strain and vascular disruption will require endochondral ossification (Fig. 6), while fractures with little to no interfragmentary strain or vascular disruption can be healed without a cartilage intermediate through direct intramembranous ossification (Fig. 5).

In theory, fractures that are perfectly realigned with rigid fixation heal through primary bone healing. At the other end of the spectrum, a fracture with a large, hypoxic osseous defect and disproportionate interfragmentary strain requires a cartilage intermediate and will heal almost entirely through endochondral ossification. In reality, the majority of fractures will fall between these ossification extremes and employ a combination of intramembranous and endochondral ossification, also referred to as secondary bone healing (Fig. 6). For example, even in the best approximated fracture there are areas of avascular necrosis and increased strain that stimulate chondrocyte development and result in some degree of endochondral ossification. Likewise, even in a widely displaced fracture, following reduction there are regions of intramembranous bone formation at the periphery of the fracture fragments where periosteal blood supply remained intact [16].

Clinically, preference is often given to primary bone healing over secondary bone healing, with the idea that achieving perfect reduction and minimal strain across a fracture site to support intramembranous ossification, without endochondral ossification, is superior. This belief was introduced in 1949 by Dr. Robert Danis in *Theorie et Pratique de I’Osteosynthese*, where he suggested that “no periosteal or endosteal callus should ever be apparent,” in fact he considered the fracture callus to be a pathological formation, development of which should be avoided [60]. In some areas of the body, particularly around articular surfaces, a precisely anatomic reduction is essential for the longevity of the functioning joint. However, it is now known that, away from articular surfaces, attempting to force primary bone healing in areas without adequate blood supply is not superior, but, in fact, can be inferior, and can risk impaired fracture repair and iatrogenic complication [61]. In strict terms of achieving bony union, there is no superiority of primary bone healing over secondary bone healing, merely two different tools tailored for different fracture situations. Instead, the relative “pros” and “cons” of both types of stabilization must be weighed with particular focus on patient comorbidities, concurrent injuries, status of surrounding soft tissues, technical challenges of each surgical approach, and best clinical outcome for the patient.

Innovation opportunity: An in vivo measurement of strain across the fracture site would benefit surgeons within the operating room in applying the most appropriate construct targeted to a certain strain value. A similar dynamic strain output during the several weeks of recovery would trace the patient’s course and allow for directed adjusted of construct stiffness and warn against impending fracture delay or non-union.

## Vascularity in Fracture Healing

The importance of vascularity for bone health was known many decades ago when histologic assessment showed that osteocytes were never more than 200 μm away from a vessel [62]. But the specific revascularization pattern of a healing fracture was debated throughout the twentieth century by physician-scientists with particular focus on the relative contribution of periosteal and intramedullary vessels. While the first theory highlighted “outside-in” or centripetal flow from the periosteum to the fracture site [63], this was challenged later by the contribution of intramedullary blood supply to fracture revascularization, leading to the centrifugal model [64–67]. This initial disagreement was ultimately resolved in the 1960s when Rhinelander demonstrated that revascularization was dependent on the type of fracture model used and both periosteal and intramedullary vasculature are significant contributors to fracture healing [68–70]. Knowledge of the patterns of revascularization is required to understand how an orthopedic construct can affect the remaining vascularity during fracture repair.

**Fig. 7:**
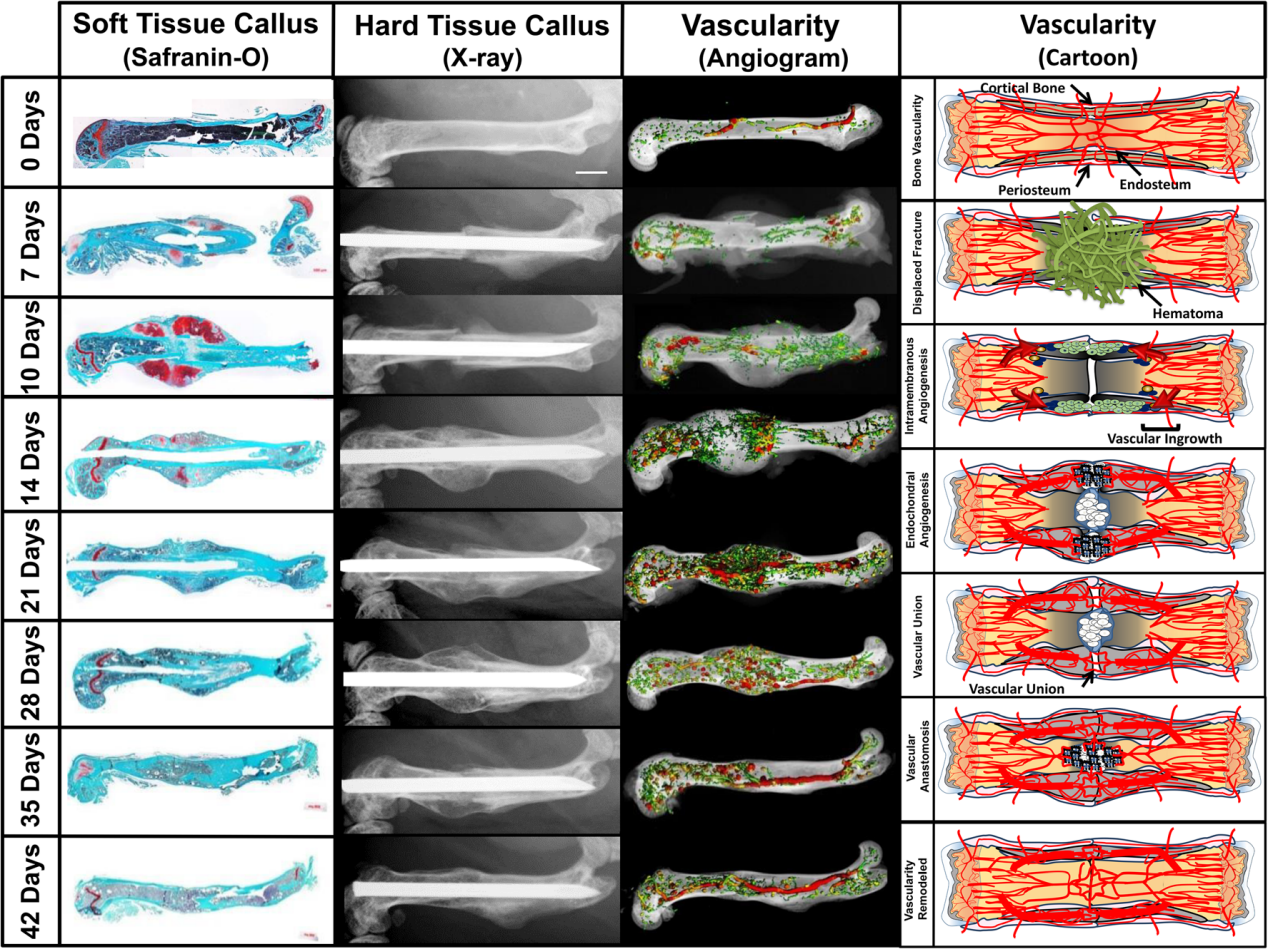
Temporo-spatial fracture repair and angiogenesis. In a murine diaphyseal fracture model, the production and resolution of soft tissue callus, hard tissue callus, and vascularity are highly correlated with one another. Safranin-O staining, radiographs, and angiograms of fractured femurs demonstrate the temporal and spatial development of the fracture callus and associated vasculature. Seven days post fracture (7-DPF), the diaphyseal intramedullary vasculature remains disrupted by regional hematoma, resulting in an avascular femoral segment flanked proximally and distally by intact intramedullary vasculature and shunting blood to the periosteum. Radiographic and histopathologic examination shows formation of a cartilaginous soft tissue callus without evidence of osteoid formation within this avascular zone. The soft tissue callus rapidly enlarges to its maximal size by 10-DPF. Simultaneously, hard tissue callus is initially formed via intramembranous ossification at the extreme proximal and distal aspects of the fracture site, where the periosteum inserts on unaffected adjacent cortical bone. This process occurs in conjunction with the formation of small highly branching extramedullary vessels recruited by cells in the periosteum expressing VEGF-A (10-DPF). As hard tissue callus replaces soft tissue callus (14-DPF), it is accompanied by an expansion of newly formed vasculature. The regions of vascular expansion begin at the proximal and distal aspects of the fracture site and migrate centrally toward the soft tissue callus, directed by the ordered release of VEGF by hypertrophic chondrocytes. Vascular ingrowth continues until anastomoses are developed, coinciding with complete dissolution of soft tissue callus and formation of bridging hard tissue callus (21-DPF). Following a vascular anastomosis and bridging of hard callus across the fracture site, the fracture callus remodels back to within the original cortices coinciding with the vasculature returning to larger vessels with reduced branching (28–42-DPF)

**Fig. 8:**
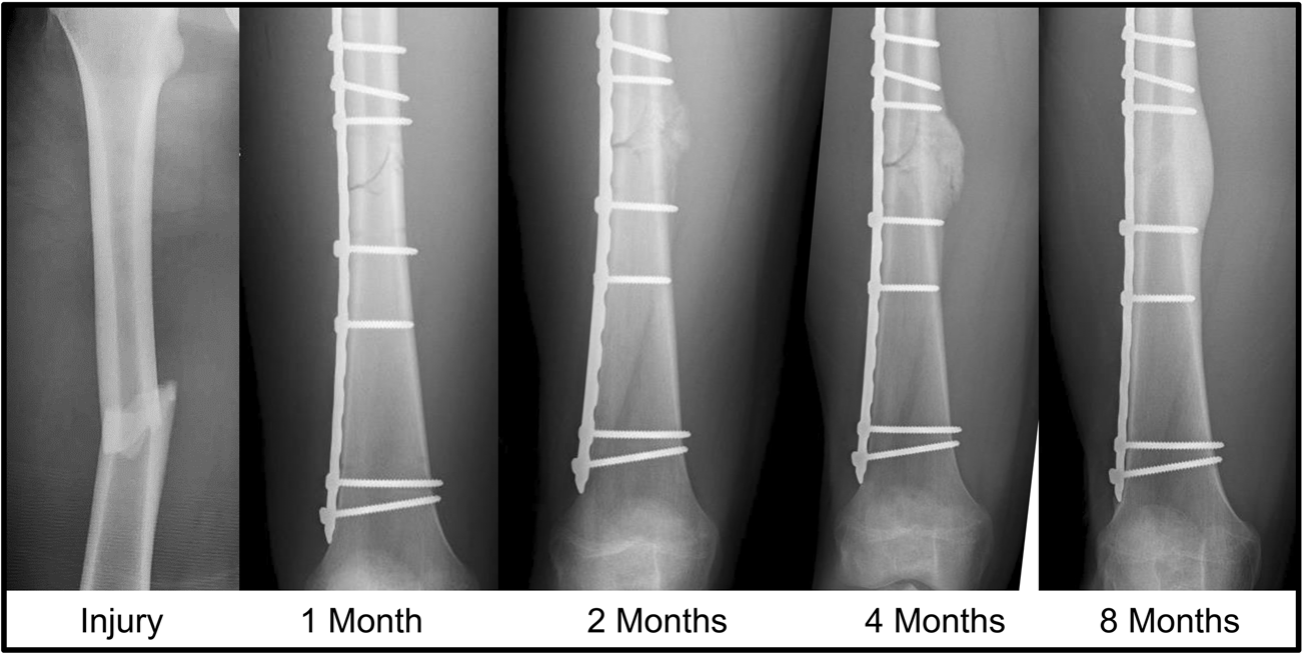
Radiographs as “angiograms.” These radiographs depict a healing femur fracture in a young adult treated with open reduction and internal fixation with plate and screws. The hardware provides greater stabilization on the ipsilateral side of the fracture and more chondroid soft tissue callus is required on the contralateral fracture side for equivalent stabilization. The lack of motion on the side of the plate coupled with the compression of the fracture prevents callus formation. The hazy soft tissue callus become radiopaque as it is replaced by hard tissue, which is definitive evidence of vascular ingress to the area

As described above, vascularity is a primary driving force behind both intramembranous and endochondral ossification. The production and resolution of soft tissue callus, hard tissue callus, and vascularity are highly correlated with one another (Fig. 7). Specifically, soft tissue callus volume expands rapidly soon after a fracture to promote initial stabilization; however, this matrix diminishes at the same rate that hard tissue callus and blood vessel volume expand. Hard tissue callus and vessel volume expand until union occurs, then they too begin to reduce during the remodeling phase toward pre-injury levels. Blocking angiogenesis with endostatin in a murine fracture model caused significantly higher callus formation and inhibited callus remodeling [71]. This re-demonstrates that fracture healing cannot proceed until all previous steps are completed. In the endostatin model, a large avascular cartilage callus reduced strain but the vascular dependent processes—conversion to hard tissue callus and hard callus remodeling—were inhibited. This dynamic relationship between soft tissue callus, hard tissue callus, and vessel growth enables re-examination of fracture X-rays as inferred angiograms, not just evidence of osteogenesis (Fig. 8) [16]. The clinical impact of deficient vascularity is evident in that many of the most common conditions associated with fracture non-unions—diabetes, smoking, advanced age—all have significant vascular components.

Innovation opportunity: a minimally invasive means of investigating vascularity at the time of fracture would aid surgeons in applying the appropriate construct by identifying the borders of intact blood supply around a fracture. In combination with an intraoperative fracture strain measurement, this would create the most ideal fracture construct application aid.

## Summary—The Unified Model of Fracture Repair

When an initial trauma causes a fracture, there are five principle problems that must be resolved: bleeding, risk of infection, disproportionate strain, bone hypoxia, and inability to bear weight. The body’s APR responds to the stimuli generated by the injury (cytokines, hypoxia, uncontrolled strain) by first acting to contain bleeding and infection, and subsequently by synthesizing a temporary chondroid soft tissue callus to control strain. Once strain across the fracture site is appropriately reduced, chondrocytes direct vascular ingrowth to restore vascular union and eliminate hypoxia in the injury site. Having re-established vascular continuity across the fracture site, osteoblasts can now fill in the defect and create an immature hard callus that can bear weight, albeit inefficiently. Finally, the initial hard callus is remodeled based on repetitive load bearing into a structurally and metabolically efficient construct.

The amount of strain and vascularity at a fracture site determines the type of ossification. Therefore, the type and method of fixation applied by the orthopedic surgeon modifies the strain experienced at the fracture site and the type of ossification that follows. A complete understanding of this unified theory of fracture repair, particularly the roles of vascularity and strain, will aid surgeons as they decide how to best treat each fracture they encounter.

## Applied Fracture Fixation Principles

### Considering Ossification Type

Building upon the foundation of the unified model of fracture repair, we will now apply these principles to clinical considerations that must be taken when treating a fracture. First, as discussed above, strain and vascularity at the fracture site fundamentally dictate the type of ossification that follows. Given that the strain experienced by a fracture is highly dependent on the fracture pattern, if strain is too high (> 100%), there will be no granulation tissue because cells cannot survive such distorting forces [72]. However, levels below 100% but above 10% are still too unstable for secondary bone healing [73–75]. Strain of less than 10% permits secondary bone healing (vascular ingress and the production of woven bone), while strain less than 2% allows for primary bone healing. Thus, both overly rigid and exceedingly flexible constructs can lead to impaired fracture repair and the development of non-union [76].

The clinical approaches for modulating strain have increased as the knowledge of the physiologic mechanisms and surgical outcomes of fracture healing has increased [77, 78]. We now know that the type of strain has an important effect on bone healing, such that interfragmentary compression is widely known to promote healing [79–81]. However, the timing of the load is important as early compression and excessive compressive forces can inhibit healing [79]. On the other hand, tensile loads are more likely to prevent fracture healing and significantly high tensile loads can even lead to cortical resorption. However, low tensile loads, like other types of strain, may actually promote callus formation [73].

The role of shear strain in fracture healing is more controversial. While it is difficult to precisely compare tissue strain across studies, it appears that shear strain, in general, inhibits healing [82, 83], though some studies suggest that low levels of shear strain may be beneficial [84], especially in conjunction with compression. Regardless of the type of strain, increased strain at the fracture site necessitates increased callus size to stabilize the fracture. Studies employing fixation with less rigidity have shown that both a larger soft and hard tissue callus results [85].

As reviewed earlier, when primary bone healing is desired, a surgeon may apply fixation with the goal of absolute stability. This can be accomplished by using plates and screws in a manner that generates compression across the fracture site, minimizing strain. Fractures fixed in this way heal through intramembranous ossification. Technical pearls, like bending a plate before application to generate compression across the far cortex, are used to further promote primary bone healing across the fracture site.

**Fig. 9:**
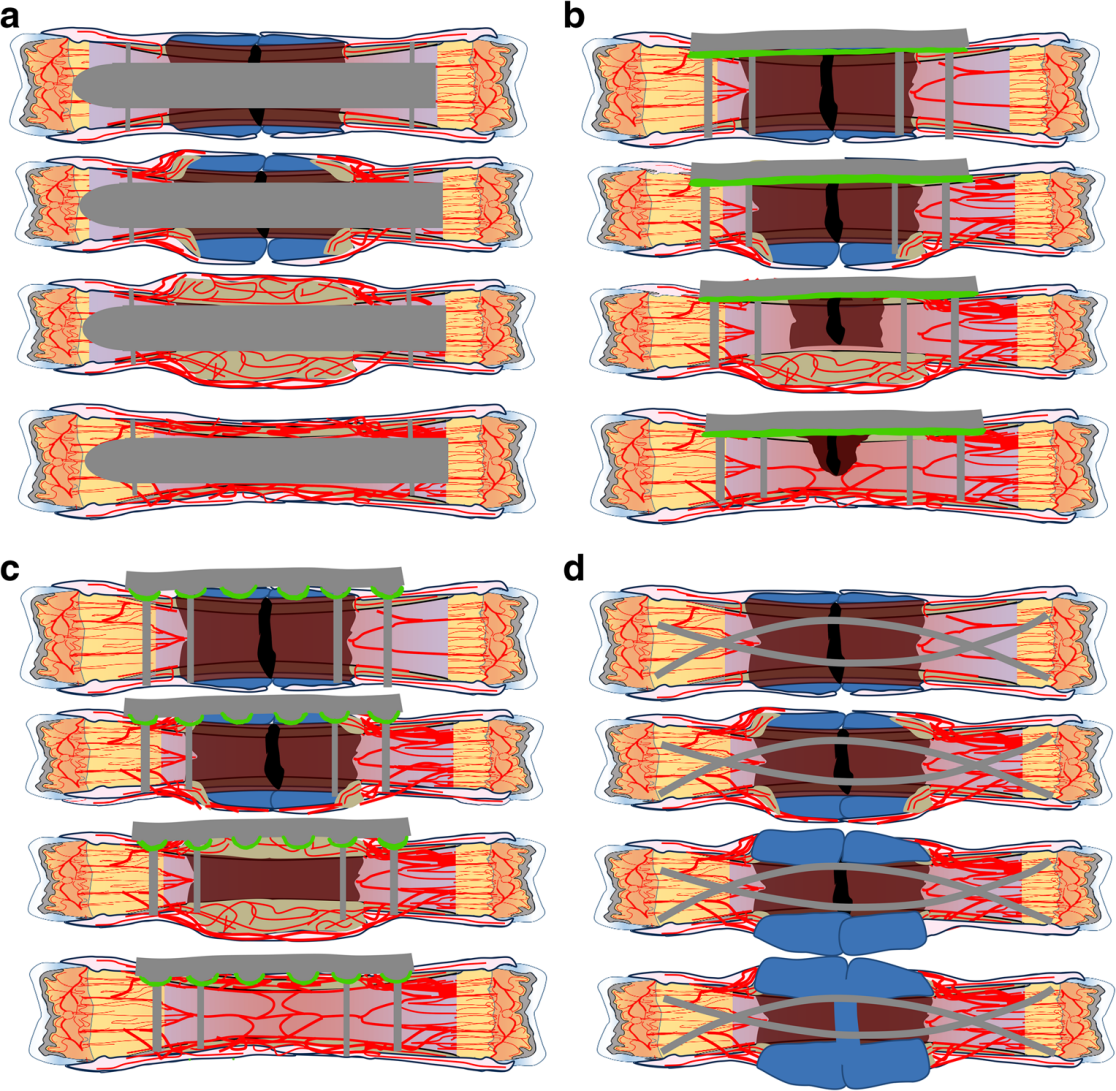
Fixation methods. (**a**) Intramedullary nailing disrupts the medullary vasculature yet leaves periosteal vessels intact. It also allows for limited motion, promoting chondrocyte proliferation. This combination enables robust callus formation that is highly vascular. (**b**) Plate and screw fixation of fractures provides rigid fixation, limiting callus formation and causing little disruption of the intramedullary vasculature. However, the compression of the plate against bone disrupts extramedullary vasculature and can cause hypoxia under the plate. (**c**) Limited contact plating aims to provide rigid fixation, leaving remaining intramedullary vasculature intact while also causing as little disruption of extramedullary vasculature as possible. This theoretically enables improved fracture healing with limited callus formation compared to full contact plating. Periosteum disrupted by these two plating methods is marked in green. (**d**) When fixation is inadequate, as is often the case of flexible nailing of an adult fracture, chondrocytes proliferate to try to reduce the strain that has not been adequately treated. However, the strain and motion may be too great for the chondrocytes to stabilize, preventing chondrocyte hypertrophy and bony union, leading to pseudarthrosis

On the other hand, there are settings where absolute stability and primary bone healing are not feasible or desired. Specifically, absolute stability increases the risk of nonunion if (1) reduction is not achieved, (2) the fracture has significant avascular segments, or (3) the application of the plate creates significant avascularity [61, 75]. Overly rigid fracture fixation in this hypoxic setting will prevent stimulus for callus formation and delay bone healing (Fig. 9) [86]. Absolute stability in these cases should be avoided.

Severe comminution, extensive soft tissue injury, or a fracture in a poorly vascularized region of bone commonly result in a hypoxic fracture healing environment regardless of fixation method. In such a case, when a requirement for intramembranous ossification is lacking, a surgeon can still promote successful bony union by creating an optimal environment for chondrocyte differentiation and endochondral ossification. Understanding how to create this environment through thoughtful application of implants is critical in fracture care. Perren described the concept of “biological plating” and “biological internal fixation” where surgeons use internal plates to bridge segments of comminuted bone instead of attempting perfect reduction [61]. In this way, he decreased rigidity across the fracture site, and permitted micromotion to promote chondrocyte development and soft tissue callus formation [87].

### Plating

When plating, close attention must be paid to preserving the vascularized soft tissues and periosteum at the level of the fracture. Preserving the periosteum, a source of stem cells and growth factors for fracture healing, allows surgeons to limit avascularity and to maintain an important mediator of subsequent revascularization [88–90]. These concepts have been instrumental in implant development, and modern plates reflect this focus on respecting the soft tissues and periosteum. Limited contact plates employ reefing to preserve underlying periosteum, with bony contact occurring only in the area surrounding screw holes [91]. Percutaneous plating techniques, such as in minimally invasive plate osteosynthesis (MIPO), have been designed to limit dissection to only what is needed for plate application, thus maximally preserving the vascularity to the tissue surrounding a fracture [92]. A number of implants have been developed with this aim, and all emphasize the importance of preserving vascularity and soft tissues while providing appropriate stability (Fig. 9).

### Intramedullary Nailing

Intramedullary nailing is an effective treatment for many diaphyseal fractures and promotes many of the previously discussed elements of fracture healing. Nails are used as load-sharing devices that provide relative, as opposed to absolute, stability. Permitting micromotion within the fracture encourages chondrocyte development and subsequent endochondral ossification. Additionally, nailing prevents iatrogenic devitalization of the periosteum near the fracture.

However, the effect of reaming on a bone’s vascularity is an important consideration when inserting an intramedullary nail. While reaming is done at a cost to the endosteum, this deleterious effect is limited and compensated for by the periosteal blood supply. Cortical ischemia and necrosis caused by reaming is followed by both reconstitution of the medullary blood supply and reversal of the centripetal flow to centrifugal from the intact periosteum. Understanding this relationship is important because an insult to both the endosteal and periosteal blood supply (e.g., open reduction and nailing) would be expected to cause a more significant vascular insult at the level of the fracture and a commensurate increase in the time to revascularization and bony union. Similarly, a larger diameter nail made possible with reaming is more rigid and improves stability, but may devitalize more cortical bone and prolong the regeneration of the medullary blood supply [93]. One must balance the need for stability with devitalization of a bone’s vasculature when choosing the size of an intramedullary nail. Often, using the smallest nail that provides the required amount of stability is preferred over a rigid, complete isthmic fitting nail.

Numerous clinical studies have attempted to optimize fracture healing through modification of one or more of the parameters that impact the stability and initial soft tissue insult of a nail construct (nail size, locking, nail composition, or pre-reaming) with varying results [94–99]. It is the authors’ opinion that the ideal nail, here termed the “biological nail,” is likely a dynamic one where stability can be altered during the course of a fracture healing to promote each desired biological process of healing. For example, it could permit relatively more micromotion during the development of the soft tissue callus thus bolstering mesenchymal cell differentiation into pre-hypertrophic chondrocytes [3–5]. Once sufficient soft callus has formed, increasing the nail’s stability would allow for the large number of chondrocytes to hypertrophy, release VEGF, and promote robust vascular ingress and subsequent ossification. As an example, one study created rudimentary “biological nails” by using sequential magnetic compression of the nail to heal humerus fractures at risk for non-union. The study reported excellent rates of healing all with the formation of large fracture calluses [99]. While studies like these are promising, further research and innovation is required to determine which mode of dynamization best supports the biomechanical and biological needs of every fracture.

## Conclusions

Despite advances in orthopedic care, fractures remain an important public health concern due to their frequent and serious consequences. This review highlights the five problems that accompany every fracture (bleeding, susceptibility to infection, disproportionate strain, bone hypoxia, and inability to bear weight), their associated complications, and the body’s stepwise approach to resolve them (the APR). We have synthesized a wide range of basic science and clinical studies to put forth a unified, rule-based working model of fracture repair with two goals in mind.

First, this model provides scientists with essential variables to control or monitor—hemorrhage, infection, strain, vascularity, ossification—in any basic or translational science endeavor exploring fracture repair. Viewing experiments through the lens of the APR and controlling these variables in the laboratory will translate into a host of clinically applicable tools. The most pressing basic science questions in fracture repair currently are (1) delineating which cells, cytokines, and matrix proteins are essential in the fracture hematoma over the course of its evolution; (2) the precise origin and function of progenitor cells throughout fracture healing; (3) models of strain modulation through the course of fracture healing; and (4) pharmacologic methods of augmenting vascularity in clinically relevant models of old age, diabetes, heart disease, and endothelial dysfunction.

Second, this model informs clinicians of the essential principles for fracture management. Specifically, treating every fracture as series of problems that require specific intervention, and recognizing appropriate fracture management requires resolution of one step before transitioning to the next. Rapid resolution of hemorrhage with fracture reduction and surgical intervention if necessary is the foremost concern. Then stabilization to reduce strain, recognizing the insertion of any orthopedic construct has both immediate and long-term biological consequences, and these consequences are directly related to the construct’s biomechanical properties. Finally, supporting vessels and stem cell division for vascular and bony union. Essential questions for the future of clinical fracture care include (1) a viable method of measuring biological potential, strain, and vascularity in vivo during fracture stabilization; (2) development of a dynamic modulating strain construct that can be adjusted throughout the repair process; (3) best practices for optimizing fracture healing in patients with deficient vascular function or a reduced biological potential to produce bone; and (4) development of clinically viable chondroid autograft or allograft products for supplementing at-risk fracture fixations.

Innovative efforts will lead to the development of a variety of interventions that address each of the problems of fractures to create the optimal biological and biomechanical healing environment every time. Perhaps one day, even in the most severe of cases, perfect fracture healing will become an expectation.
